# Effect of gamma irradiation on physicochemical properties of stored pigeon pea (*Cajanus cajan*) flour

**DOI:** 10.1002/fsn3.50

**Published:** 2013-07-12

**Authors:** Oluwaseun P Bamidele, Charles T Akanbi

**Affiliations:** Department of Food Science and Technology, Obafemi Awolowo UniversityIle-Ife, Osun State, Nigeria

**Keywords:** Functional properties, gamma irradiation, pigeon pea flour, proximate composition, sensory evaluation

## Abstract

The effect of gamma irradiation at various doses (5, 10, 15, 20 kGy) was observed on pigeon pea flour stored for 3 months on proximate composition, functional properties, and peroxide value. Sensory evaluation was also carried out on bean cake (*moinmoin*) made from nonirradiated and irradiated pigeon pea flour. The results showed that stored gamma-irradiated samples had significantly lower (*P* < 0.05) value of protein and little or no effect on moisture content. There were slight decreases in crude fiber and ash content of the irradiated samples compared with the control sample. The result of functional properties of the irradiated flours showed slight increase in water absorption capacity, swelling capacity and bulk density. The peroxide value of crude oil increased significantly with dose increases for the period of storage. The sensory evaluation of *moinmoin* samples prepared from irradiated pigeon pea flour showed no significant difference from the *moinmoin* sample prepared from nonirradiated flour. It can be concluded that gamma irradiation can extend the shelf life of pigeon pea flour.

## Introduction

In tropical and Third World countries, an acute shortage of protein-rich foods due to population explosion and cost-effectiveness of animal-based protein has laid an overwhelming interest to explore underutilized proteinaceous wild leguminous plants. Underutilized wild legumes are of immense value due to low cost, high nutritional value, and presence of health-promoting bioactive compounds. Pigeon peas are nutritionally important, as they contain high levels of protein and the important amino acids methionine, lysine, and tryptophan. In combination with cereals, pigeon peas make a well-balanced human food. Pigeon peas are very drought resistant and can be grown in areas with less than 650 mm annual rainfall (Faris et al. 1987). The usage is many as it can be combined with rice and other foods to make balanced diets. So many problems facing the utilization of pigeon pea seed has been solved. Researchers have provided a way out to the problems of cooking and preservation of pigeon pea seed by dehulling and fermentation process which improve the protein content and that of its texture (Fasoyiro et al. 2006, 2009). Radiation processing of plant produce has become an important physical preservation method to overcome the international quarantine barriers and to increase the safety and shelf life of the product, mainly by the elimination of spoilage microflora (Diehl 1995). Irradiation, besides being successful in decontamination and disinfestations, is also known to improve the quality of fresh plant produce (e.g., legumes, seeds, and spices) requiring long-term preservation (Farkas 1998; Hayashi 1998). Radiation processing is also efficient in decreasing or eliminating some of the antinutritional factors in seeds (Siddhuraju et al. 2002; Brigide and Canniatti-Brazaca 2006; Bhat et al. 2007). Of late, with the increased database and scientific evidence, health-conscious consumers are willing to use irradiated foods for safety concerns (Hunter 2000). The effect of gamma irradiation on stored food samples is of great importance, as its knowledge will help in knowing how long the food sample can be stored without affecting the nutritional component. Hence, the present studies were undertaken to find out the effects of γ-rays (low and high dose) on the nutritional composition of stored pigeon pea flour; it also aimed to investigate the nutritional and functional properties of raw and irradiated pigeon pea flour stored for 3 months.

## Materials and Methodology

Pigeon pea was purchased at Odori market in Iwo, Osun State and gamma irradiation process at Energy Centre in Abuja at different doses (5, 10, 15, and 20 kGy). The samples were exposed to gamma rays generated by a cobalt-60 source (Gammacell 220; MDS Nordion, Ottawa, Canada) following the procedures described by Helinski et al. (2008) with a dose rate of ca. 2 Gy/min at 25°C and normal relative humidity. Double side irradiation (exposure to both sides) was performed for uniform dose delivery. A dosimetry system was used to measure the dose received by the batch based on the Gafchromic HD-810 film (International Specialty Products, NJ; FAO/IAEA/USDA 2003). Three dosimeters were included with each batch of pigeon pea seeds and read after irradiation with a Radiachromics reader (Far West Technology Inc., Goleta, CA). All experiments were repeated three times and three replicates of each sample type were irradiated. The irradiated samples were milled with a hammer mill and packaged inside low-density polythene bags. Various analyses were carried out on it immediately after milling and the same analyses were carried out at 2 weeks' intervals for 3 months. The results were compared with those of nonirradiated samples. Various samples were also used to make *moinmoin* to check the level of acceptability by consumers.

### Proximate composition

The level of moisture, crude protein, crude fat, ash, crude fiber, and carbohydrates of the irradiated (5, 10, 15, and 20 kGy) and nonirradiated pigeon pea flours was determined by the use of manual methods as outlined by AOAC (2000).

### Determination of peroxide value

Peroxide values of the petroleum ether extract of pigeon pea flour were determined as described in the methods of AOAC (2000). Exactly 5 g sample of oil was weighed into a 250 mL Erlenmeyer flask and then 30 mL acetic acid-chloroform solution (3:2) solution. The flask was swirled until the sample was dissolved and 0.5 mL saturated potassium iodide (KI) solution was also added. The solution was allowed to stand with occasional swirling for 1 min and then 30 mL distilled water was added. The solution was titrated with 0.01 N sodium thiosulfate (Na_2_S_2_O_3_) until the color changed to light yellow. Exactly 0.5 mL of 1% soluble starch indicator was added. The blue solution formed was titrated with more sodium thiosulfate until the blue color disappears.


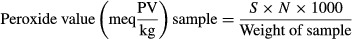


where *S* = mL of Na_2_S_2_O_3_ and *N* = 0.01 of sodium thiosulfate.

### Functional property determination

#### Water absorption determination

Water absorption was determined by the modified centrifuge method of Lin and Zayas (1987). Each sample (2.0 g) was transferred into a lagged 50 mL centrifuge tube and weighed (*W*_1_). Exactly 30 mL of hot distilled water (70°C) was added to each sample, simultaneously washing down the inside of the centrifuge tubes using a glass stirring rod; the sample and the water was mixed for 30 min. The suspension was allowed to rest for 10 min; the flour adhering to the side of the centrifuge was scrubbed down with a glass rod to prevent it from drying. An additional 10 mL of hot distilled water was used to wash the sample adhering to the stirring rod into the sample. The suspension was centrifuged at 1165*g* for 25 min at 50°C. The tube was cooled in a desiccator and weighed (*W*_2_).


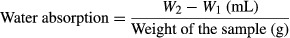


#### Bulk density determination

Bulk density was determined using the gravimetric method as described by Okaka and Potter (1976). The sample (10 g) was weighed into a 25 mL graduated cylinder. The cylinder was gently tapped 10 times against the palm of the hand. The bulk density was expressed as the sample per volume occupied by the sample.

#### Swelling capacity determination

It was determined by modification of the Lin and Zayas (1987) method. Each sample (2 g) was dispersed in 40 mL distilled water. The resultant slurry was heated at a temperature of 70°C for 30 min in a water bath, cooled to room temperature, and centrifuged at 598*g* for 30 min. The supernatant liquid was decanted and the centrifuge tube was placed in a hot air oven and dried for 25 min at 50°C. The residue was weighed (*W*_2_). The centrifuge tube containing the sample alone was weighed prior to adding distilled water (*W*_1_).

### Sensory evaluation of products from pigeon pea flours

**Figure 1:**
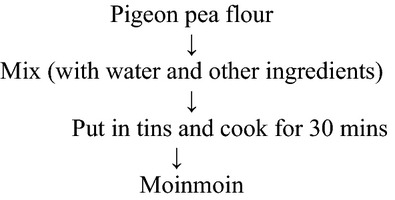
Flow chart showing *moinmoin* preparation (Fasoyiro et al. 2010).

The flours were processed into *moinmoin* as shown in Figure 1. These were compared with *moinmoin* from nonirradiated flour using the traditional method of reconstituting the flour into paste. Samples were coded and presented as random numbers to 20 panels of judges to test for the following attributes: appearance, color, flavor, taste, and overall acceptability. The panelists were provided with a mouth rinse in between each tasting. The attributes were scored using a 9-point hedonics scale, where 9 equals like extremely and 1 equals dislike extremely. The *moinmoin* recipe was: Pigeon pea flour, salt, water, vegetable oil and Flavourant.

### Statistical analysis

Each determination was carried out in triplicate and the mean taken. Data obtained from the physicochemical and sensory analysis were subjected to analysis of variance (ANOVA) and the means were separated by lowest standard deviation test (SPSS 16.0, 2008). The significant level was accepted at 5%.

## Results and Discussion

### Proximate composition

**Figure 2:**
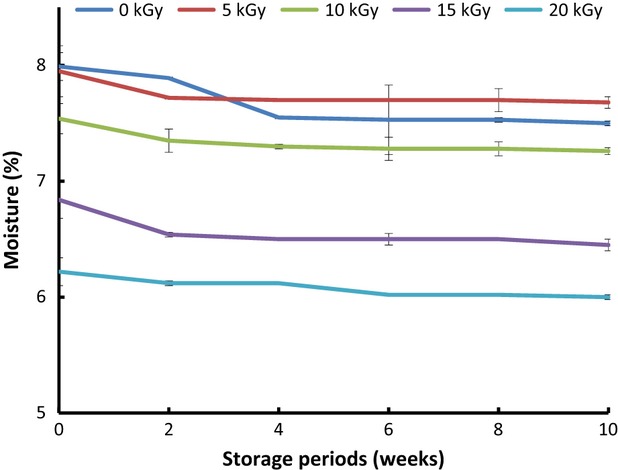
Effect of gamma irradiation on moisture content of pigeon pea flour (%) during storage.

**Figure 3:**
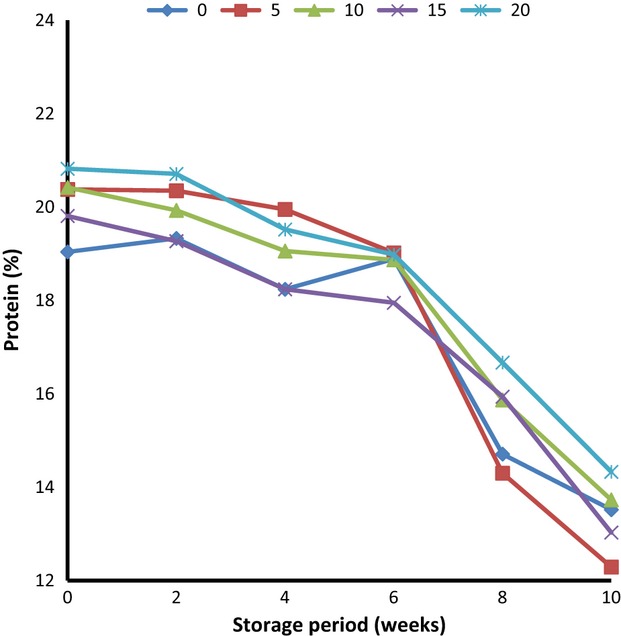
Effect of gamma irradiation on protein content of pigeon pea flour (%) during storage.

The variation in the results of moisture, protein, crude fat, crude fiber ash, and carbohydrate content during storage of irradiated and nonirradiated pigeon pea flour is presented in Figure 2. The initial moisture content of the tested flour ranged from 6.22% to 7.99% before storage, but decreased to 6.00% to 7.50% after 3 months of storage. A slight but insignificant difference was observed (*P* > 0.05). These results were similar to those obtained in irradiation of rawa (Rao et al. 1994). There was a decrease in protein content as show in Figure 3 throughout the period of storage. Changes in protein fraction may be related to some cross-linking or aggregation of protein as a result of gamma irradiation affecting nitrogen solubility (Ciesta et al. 2000). ANOVA showed a significant difference among the various samples irradiated with different doses (*P* < 0.05) throughout the period of storage. This is consonant with the report of Imdad et al. (2010) on palm dates at gamma dose of 20, 50, 100, 200, and 300 rads. Their result showed a decrease in protein content for the first 3 months of storage. Also, Nahla et al. (2009) reported a decrease in the protein content of dry bean irradiated at gamma doses of 1, 1.5, and 2 kGy.

**Figure 4:**
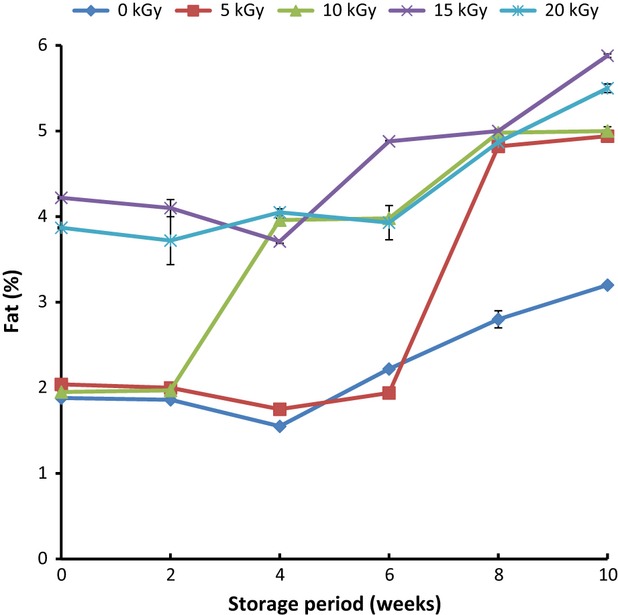
Effect of gamma irradiation on crude fat of pigeon pea flour (%) during storage.

There was a slight increase in crude fat content for the first 6 weeks of storage, while the increase was significant for the last 6 weeks (see Fig. 4). Gamma dose 15 kGy showed the highest increase all through these periods. Nahla et al. (2009) also reported an increase in fat content of dry bean irradiated at doses of 1, 1.5, and 2 kGy. The increase may be due to the effect of gamma irradiation on the small molecule of the flour which is broken down during storage. Analysis of the result showed a significant difference (*P* < 0.05).

**Figure 5:**
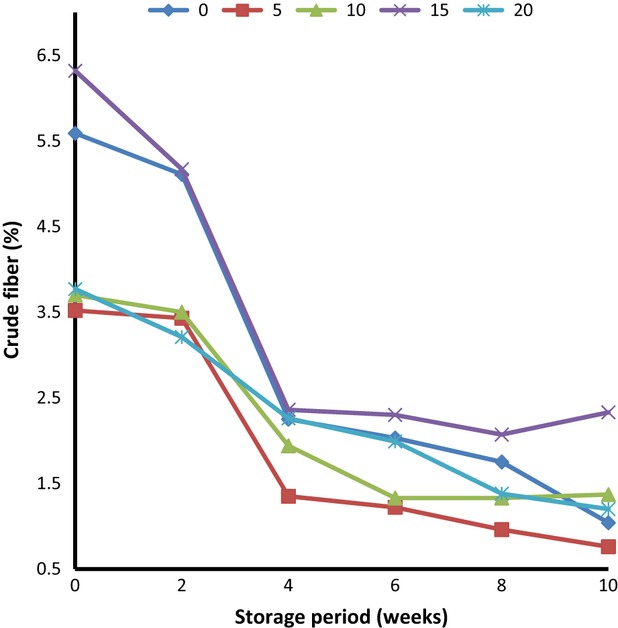
Effect of gamma irradiation on crude fiber of pigeon pea (%) during storage.

**Figure 6:**
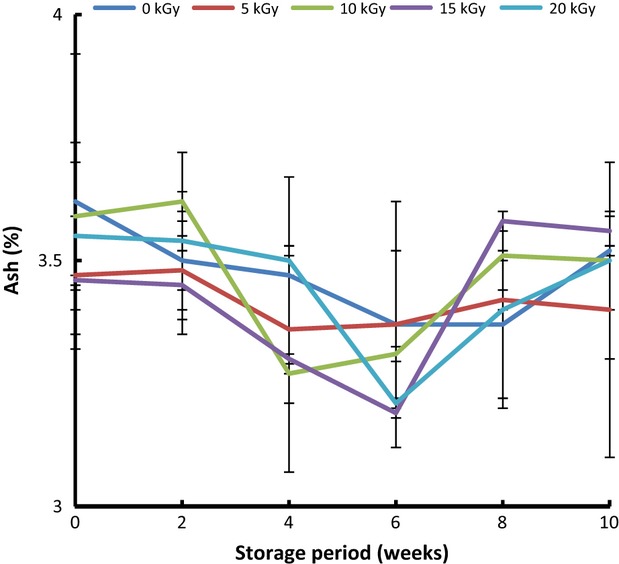
Effect of gamma irradiation on ash content of pigeon pea flour (%) during storage.

Figures 5, 6 show the results of crude fiber and ash content of the stored samples. Both crude fiber and ash content decreased throughout the period of storage, although in ash the pattern of decrease was irregular. The pattern was also reported by Nahla et al. (2009) and Imdad et al. (2010) on crude fiber contents of dry irradiated beans and palm date, respectively. Bhat et al. (2009) reported a decrease in crude fiber content of lotus seed flour irradiated at a dose range of 0–30 kGy. Reduction in crude fiber is conditionally appreciated in those that do not have diseases like obesity because it traps less protein as well as carbohydrate, but it is well appreciated in those with such diseases (Balogun and Fetuga 1986). Bhat et al. (2008) also reported a decrease in ash content of velvet bean seed exposed to gamma irradiation at a dose range of 0–30 kGy.

**Figure 7:**
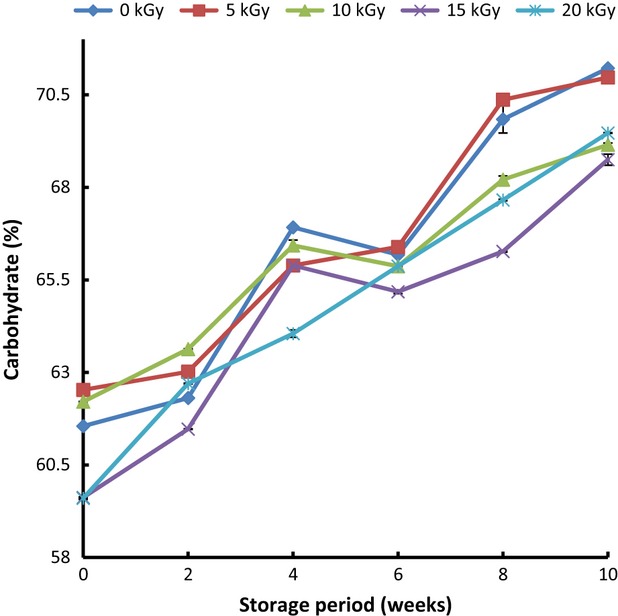
Effect of gamma irradiation on carbohydrate content of pigeon pea (%) during storage.

The carbohydrate content of all the samples showed an increase throughout the period of storage (Fig. 7), although the increase was slight. The least increase in carbohydrate content was observed at dose 15 kGy and the highest at dose 0 and 5 kGy. The analysis showed no significant difference (*P* > 0.05) at weeks 7 and 9 but a significant difference (*P* < 0.05) in the remaining weeks. An increase in the carbohydrate content of irradiated samples was also observed by other researchers (Bhat et al. 2008; Nahla et al. 2009; Imdad et al. 2010).

### Effect of gamma irradiation on functional properties of pigeon pea flour

Tables 1, 2, 3 show the results of the effect of gamma irradiation on the functional properties of pigeon pea flour. The results showed a slight increase in water absorption capacity, swelling property, and bulk density of the flour. These observations are supported by those of Bhat et al. (2009) on irradiated lotus seed flour and those of Nahla et al. (2009) on irradiated dry beans. The analysis showed no significant difference (*P* > 0.05).

**Table 1:** Effects of gamma irradiation on water absorption capacity of pigeon pea flour (mL/100 g)

	Storage period (weeks)					
	
Dose (kGy)	0	2	4	6	8	10
0	1.88 ± 0.015b	1.84 ± 0.015a	1.73 ± 0.116a	1.70 ± 0.100a	1.77 ± 0.010b	1.74 ± 0.010b
5	1.73 ± 0.153a	1.80 ± 0.100a	1.65 ± 0.030a	1.58 ± 0.227a	1.60 ± 0.006a	1.54 ± 0.010a
10	2.01 ± 0.015bc	2.01 ± 0.015a	1.94 ± 0.036a	1.96 ± 0.035a	1.80 ± 0.100b	1.87 ± 0.010d
15	2.24 ± 0.010d	2.23 ± 0.021a	2.23 ± 0.577a	1.67 ± 0.577a	1.96 ± 0.025d	1.93 ± 0.060e
20	2.00 ± 0.010b	2.28 ± 0.065a	1.95 ± 0.042a	1.97 ± 0.010a	1.82 ± 0.106bc	1.84 ± 0.025c

Values are means and standard deviation of three determinations (*n* = 3). Means followed by the same letter within the same row are not significantly (*P* > 0.05) different according to LSD test.

**Table 2:** Effects of gamma irradiation on swelling capacity of pigeon pea flour (mL/100 g)

	Storage period (weeks)					
	
Dose (kGy)	0	2	4	6	8	10
0	2.31 ± 0.010b	2.29 ± 0.015a	2.33 ± 0.015a	2.30 ± 0.010a	2.27 ± 0.010a	2.27 ± 0.020b
5	2.54 ± 0.025b	2.54 ± 0.025b	2.50 ± 0.010a	2.47 ± 0.027a	2.40 ± 0.010c	2.60 ± 0.010c
10	2.67 ± 0.010d	2.50 ± 0.100b	2.50 ± 0.100b	2.54 ± 0.010a	2.52 ± 0.021a	2.44 ± 0.030c
15	2.29 ± 0.015a	2.27 ± 0.006a	2.28 ± 0.015a	2.67 ± 1.155a	1.97 ± 0.012a	1.90 ± 0.100a
20	2.31 ± 0.010b	2.32 ± 0.100a	2.30 ± 0.100a	2.20 ± 0.010a	2.67 ± 1.155a	1.98 ± 0.012a

Values are means and standard deviation of three determinations (*n* = 3). Means followed by the same letter within the same row are not significantly (*P* < 0.05) different according to LSD test.

**Table 3:** Effects of gamma irradiation on bulk density of pigeon pea flour (mL/100 g)

	Storage period (weeks)					
	
Dose (kGy)	0	2	4	6	8	10
0	50.31 ± 0.015ab	50.30 ± 0.100a	50.31 ± 0.020c	50.34 ± 0.015ba	50.31 ± 0.015e	50.27 ± 0.015c
5	50.20 ± 0.100a	50.27 ± 0.006a	50.27 ± 0.040bc	50.27 ± 0.010b	50.25 ± 0.010b	50.25 ± 0.015b
10	50.22 ± 0.020ab	50.21 ± 0.100b	50.20 ± 0.015a	50.25 ± 0.010a	50.21 ± 0.010a	50.20 ± 0.015a
15	50.34 ± 0.027b	50.28 ± 0.015a	50.25 ± 0.015b	50.26 ± 0.015a	50.27 ± 0.015c	50.26 ± 0.015b
20	50.27 ± 0.015ab	50.31 ± 0.015a	50.30 ± 0.015c	50.34 ± 0.025b	50.30 ± 0.010d	50.32 ± 0.015d

Values are means and standard deviation of three determinations (*n* = 3). Means followed by the same letter within the same row are not significantly (*P* > 0.05) different according to LSD test.

### Effect of gamma irradiation on peroxide value of oil

**Figure 8:**
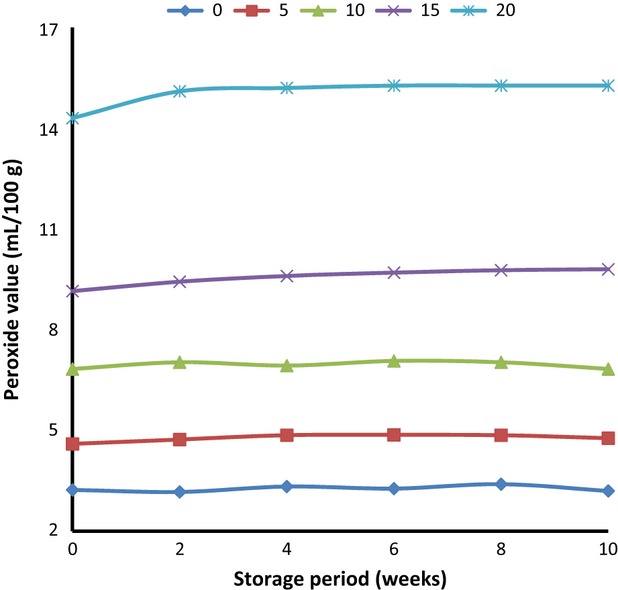
Effect of gamma irradiation on peroxide value of pigeon pea flour (mL/100 g) during storage.

The peroxide value of the crude oil of irradiated samples increased significantly with an increase in dose level. The result was corroborated by Zeb and Ahmad (2004) who reported that the acid and the peroxide value of soybean oil increased with an increase in gamma irradiation (15 and 20 kGy; Fig. 8).

### Sensory evaluation

*Moinmoin* is a traditional food product in Nigeria usually prepared from fresh cowpea paste. The appearance and color of *moinmoin* from all irradiated pigeon pea flour indicated higher scores in terms of the qualities assessed. One desirable attribute of *moinmoin* is good mouthfeel. Table 4 show the results of sensory evaluation of *moinmoin* made with irradiated pigeon pea flour. For *moinmoin*, from pigeon pea flour irradiated at 20 kGy was partially acceptable in appearance and color but fully acceptable in mouthfeel.

**Table 4:** Result for sensory evaluation of *moinmoin* prepared from irradiated pigeon pea flour

	Irradiation dose (kGy)				
	
Parameters	0	5	10	15	20
Appearance	6.467a	6.533a	6.200a	6.200a	4.867a
Color	6.133a	6.133a	6.067a	6.200a	4.600a
Taste	6.267a	6.200a	6.267a	6.067a	4.533a
Mouthfeel	6.000a	6.200a	6.333a	6.467a	4.400a
Flavor	6.133a	6.267a	6.267a	6.067a	4.467a
Overall acceptability	6.267a	6.200a	6.067a	6.000a	4.800a

Means followed by the same letter within the same rows are not significantly (*P* > 0.05) different according to LSD test.

## Conclusion

Irradiation of pigeon pea flour at a dose range of 0–20 kGy and storage for 3 months (3 months) had a significant effect on the proximate composition of the flour (moisture content, protein, crude oil, crude fiber, ash, and carbohydrate) and peroxide value. It can be concluded that the gamma irradiation dose used for storage helps in increasing the shelf life of food products. The effect of gamma irradiation on the functional properties of the flour also indicated that gamma irradiation did not affect the various properties with the storage (water absorption capacity, swelling capacity, and bulk density). The sensory evaluation of *moinmoin* made from irradiated pigeon pea flour showed no significant difference when compared with *moinmoin* made from nonirradiated flour and the peroxide value of the crude oil showed a dose increase throughout the storage period.
